# Clinical outcomes of spontaneous bacterial peritonitis due to extended-spectrum beta-lactamase-producing *Escherichia coli *and *Klebsiella *species: A retrospective matched case-control study

**DOI:** 10.1186/1471-2334-9-41

**Published:** 2009-04-12

**Authors:** Kyoung-Ho Song, Jae Hyun Jeon, Wan Beom Park, Sang-Won Park, Hong Bin Kim, Myoung-don Oh, Hyo-Suk Lee, Nam Joong Kim, Kang Won Choe

**Affiliations:** 1 Department of Internal Medicine, Seoul National University College of Medicine, Seoul, Korea

## Abstract

**Background:**

Clinical outcomes of spontaneous bacterial peritonitis (SBP) due to extended-spectrum β-lactamase-producing *Escherichia coli *and *Klebsiella *species (ESBL-EK) have not been adequately investigated.

**Methods:**

We conducted a retrospective matched case-control study to evaluate the outcomes of SBP due to ESBL-EK compared with those due to non-ESBL-EK. Cases were defined as patients with liver cirrhosis and SBP due to ESBL-EK isolated from ascites. Control patients with liver cirrhosis and SBP due to non-ESBL-EK were matched in a 3:1 ratio to cases according to the following five variables: age (± 5 years); gender; species of infecting organism; Child-Pugh score (± 2); Acute Physiological and Chronic Health Evaluation II score (± 2). 'Effective initial therapy' was defined as less than 72 hours elapsing between the time of obtaining a sample for culture and the start of treatment with an antimicrobial agent to which the EK was susceptible. Cephalosporin use for ESBL-EK was considered 'ineffective', irrespective of the minimum inhibitory concentration. ESBL production was determined according to the Clinical and Laboratory Standards Institute guidelines on stored isolates.

**Results:**

Of 1026 episodes of SBP in 958 patients from Jan 2000 through Dec 2006, 368 (35.9%) episodes in 346 patients were caused by SBP due to EK, isolated from ascites. Of these 346 patients, twenty-six (7.5%) patients with SBP due to ESBL-EK were compared with 78 matched controls. Treatment failure, evaluated at 72 hours after initial antimicrobial therapy, was greater among the cases (15/26, 58% *vs*. 10/78, 13%, *P *= .006); 30-day mortality rate was also higher than in the controls (12/26, 46% *vs*. 11/78, 15%, *P *= .001). When the case were classified according to the effectiveness of the initial therapy, 'ineffective initial therapy' was associated with higher 30-day mortality rate (11/18, 61% *vs*. 1/8, 13%, *P *= .036).

**Conclusion:**

SBP due to ESBL-EK had poorer outcomes than SBP due to non-ESBL-EK. Ineffective initial therapy seems to be responsible for the higher rate of treatment failure and mortality in SBP due to ESBL-EK.

## Background

Spontaneous bacterial peritonitis (SBP) is a major cause of morbidity and mortality in cirrhosis patients [1]. Gram negative bacilli, such as *Escherichia coli *and *Klebsiella *species (EK) are the most common cause of SBP [2,3]. Historically, early diagnosis using criteria based on ascitic polymorphonuclear leukocyte count and empirical treatment with effective antibiotics have improved the clinical outcome of SBP [4-6]. 3^rd ^generation cephalosporins, such as cefotaxime, have been viewed as the drug of choice for empirical treatment of SBP [3,6,7].

Since the 1980s, the incidence of infections due to extended-spectrum β-lactamase-producing *Escherichia coli *and *Klebsiella *species (ESBL-EK) has increased. Several studies of clinical outcomes in patients with infections due to ESBL-producing organisms have shown higher mortality and reduced rates of clinical and microbiologic response compared to infections due to non-ESBL-producing organisms [8-12]. However the impact of ESBL-producing organisms on clinical outcome has not been well described in patients with SBP and advanced liver cirrhosis. We conducted the current study to evaluate the outcomes of SBP due to ESBL-EK, based on their isolation from ascites, compared with those of SBP due to non-ESBL-EK. We also investigated the impact of ineffective initial antimicrobial therapy on outcome in patients with SBP due to ESBL-EK, and the risk factors for infection by ESBL-producing microorganisms.

## Methods

### Patients

To identify patients with advanced liver cirrhosis and SBP due to EK isolated from ascites, we reviewed the database at the clinical microbiology laboratory, and the medical records, by the diagnosis at discharge from 1 Jan 2000 to 31 Dec 2006. EK which was not susceptible to either cefotaxime or ceftazidime was considered 'suspected ESBL-EK'. This study was approved by the institutional review board by Seoul National University Hospital.

### Microbiologic methods

Most of the ascitic isolates were collected by the clinical microbiology laboratory in our hospital. Species identification was carried out by standard methods with VITEK-GNI cards (bioMerieux, Hazelwood, Mo.).

The antibiotic susceptibility of each isolate was determined by the disk diffusion method, using the criteria of the Clinical and Laboratory Standards Institute (CLSI). ESBL production was determined by the disk diffusion method according to CLSI performance standards. Briefly, we determined the diameters of the inhibition zones on cefotaxime and ceftazidime disks (30 μg each), alone and in combination with clavulanic acid (10 μg). An increase of ≥5 mm in zone diameter when either of the antimicrobial agents was combined with clavulanic acid was considered evidence of ESBL production. Two control organisms, *E. coli *ATCC 25922 and *K. pneumoniae *ATCC 700603, were inoculated in each set of tests for quality control.

### Definitions and data collection

Cases were defined as patients with advanced liver cirrhosis and SBP due to ESBL-EK, isolated from ascites. Controls were patients with advanced liver cirrhosis and SBP due to non-ESBL-EK. They were matched in a 3:1 ratio to case patients according to the following five variables: age (± 5 years); gender; species of infecting organism; Child-Pugh score (± 2) [13]; Acute Physiological and Chronic Health Evaluation (APACHE) II score (± 2) [14]. If more than three patients were selected for candidate of control, we chose three patients who had a Child-Pugh score close to that of the case patient.

We reviewed the medical records of both case and control-patients. The data collected included: age, gender, species of infecting organism, Child-Pugh score, severity of illness calculated by the APACHE II score, presentation with septic shock, presence of bacteremia, care in the intensive care unit (ICU) and antimicrobial regimen. The following conditions were also documented to identify 'risk factors' for infection by ESBL-producing microorganism: hospital stay before onset of SBP, presence of hepatocellular carcinoma, history of SBP, antimicrobial therapy within the 30 days prior to onset of SBP, neutropenia, presence of central venous catheter, indwelling urinary catheter, use of immunosuppressive agents including corticosteroid within 30 days, polymicrobial infection and invasive procedure within 72 hours prior to SBP.

Primary outcomes were the initial treatment response, and 7-day and 30-day mortality rates. The initial treatment response was assessed 72 hours after starting antimicrobial therapy and was classified as follows: 'complete response' for patients who had resolution of fever, leukocytosis and all signs of infection; 'partial response' for patients who had abatement of abnormalities in the above parameters without complete resolution; 'failure' for patients who had died or deterioration or absence of abatement of fever, leukocytosis and all other signs of infection [10].

The diagnosis of SBP was based on the combination of a positive ascitic fluid culture and a polymorphonuclear leukocyte (PMNL) count of >250 cells/μL, or on a combination of symptoms/signs of SBP and a PMNL count of >250 cells/μL [15]. Neutropenia was defined as an absolute neutrophil count below 500/μL.

'Effective initial therapy' was defined as less than 72 hours elapsing between the time of obtaining a sample for culture and initiation of treatment with an 'effective' antimicrobial agent. The antimicrobial therapy was considered 'effective' if the treatment regimen included antibiotics active in vitro, and the dosage and route of administration were in conformity with current medical standards. Cephalosporin monotherapy for ESBL-EK was considered 'ineffective', irrespective of the minimum inhibitory concentration.

### Statistical analyses

The Pearson chi-square test, Fisher's exact test, Wilcoxon signed rank test and Kaplan-Meier survival analysis were used to compare clinical variables and outcomes, as appropriate. All *P *values were two-tailed, with *P *< 0.05 being considered statistically significant. We performed univariate analysis for the relationship between risk factors and infection by ESBL-producing EK using conditional logistic regression. SPSS software version 15.0 (SPSS Inc, Chicago, IL) was used for these analyses.

## Results

### Study populations

From 1 Jan 2000 to 31 Dec 2006, 1026 episodes of SBP were identified in 958 patients. Of the 1026 episodes, 368 (35.9%) in 346 patients were diagnosed as SBP due to EK isolated from ascites. Of the 368 episodes, 32 (8.7%) were due to 'suspected ESBL-EK'. All 32 strains of 'suspected ESBL-EK' were stored, and all but one were recovered. Each 'suspected ESBL-EK' caused one episode of SBP in one patient. We performed a confirmatory test for ESBL-production on these 31 EKs. Twenty-six were confirmed as producing ESBL. These 26 patients were included in the case group and the triple number of patients was selected for the control group. Clinical characteristics including five matched-variables in these patients are shown in Table 1.

**Table 1:** Clinical characteristics of patients with spontaneous bacterial peritonitis (SBP)

Clinical characteristics	SBP due to		*P*-value
		
	ESBL-EK (n = 26)	Non-ESBL-EK (n = 78)	
Age (mean in year, SD)	59.5 ± 10.0	60.0 ± 9.7	0.799
Male (n, %)	19 (73%)	57 (73%)	-
*Escherichia coli *(n, %)	17 (65%)	51 (65%)	-
Child-Pugh score (mean, SD)	10.8 ± 1.6	10.6 ± 1.8	0.582
APACHE II score (mean, SD)	15.6 ± 3.7	15.3 ± 3.5	0.710

ESBL-EK – extended-spectrum β-lactamase-producing *Escherichia coli *and *Klebsiella *species

SD – standard deviation

APACHE II – Acute Physiologic and Chronic Health Evaluation II

ICU – intensive care unit

### Clinical outcomes

**Figure 1:**
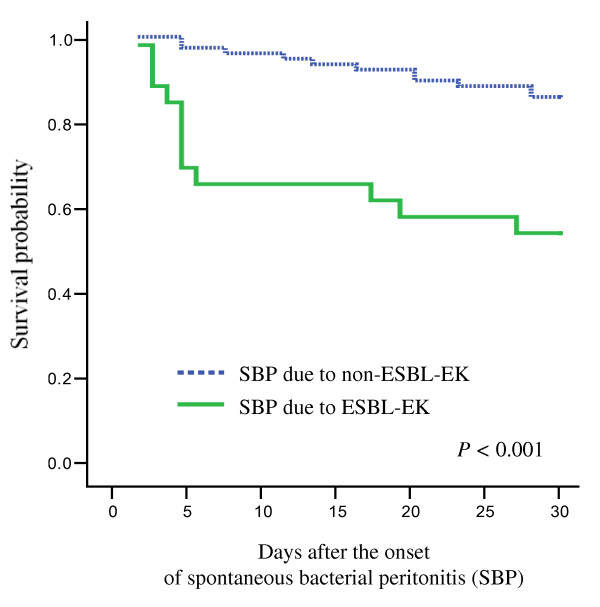
**Survival curves obtained by the Kaplan-Meier method**. Survival curves for spontaneous bacterial peritonitis (SBP) due to *Escherichia coli *and *Klebsiella *species (EK), as a function of the production of extended-spectrum β-lactamase (ESBL). The *P *values shown were calculated by the log rank test.

Clinical outcomes and survival curves for SBP are shown in Table 2 and Figure 1. The treatment failure rate of the case patients, assessed 72 hours after initial therapy, was higher than that of the control patients (58% *vs*. 13%, *P *= .006). The 30-day mortality rate of the cases was also higher than that of the controls (46% *vs*. 14%, *P *= .001).

**Table 2:** Clinical outcomes of patients with spontaneous bacterial peritonitis (SBP) due to *Escherichia coli *and *Klebsiella *species (EK), according to the production of extended-spectrum β-lactamase (ESBL)

Outcomes	SBP due to		*P*-value
		
	ESBL-EK (n = 26)	Non-ESBL-EK (n = 78)	
Initial response^†^			
Complete response (n, %)	2 (8%)	28 (36%)	< 0.001
Treatment failure (n, %)	15 (58%)	10 (13%)	0.006
7-day mortality (n, %)	9 (35%)	3 (4%)	< 0.001
30-day mortality (n, %)	12 (46%)	11 (14%)	0.001

^† ^Initial response was assessed 72 hours after the initiation of empirical treatment

Of the 26 case patients, eight (31%) had effective initial therapy with imipenem, whereas all of the control patients received effective initial therapy with 3^rd ^generation cephalosporin. Of the 18 case patients who had ineffective initial therapy, two died within 72 hours of the start of the empirical treatment. Apart for these two patients, the remaining 16 patients received antibiotics (carbapenems) effective against ESBL-EK after receipt of reports of the susceptibility tests. The clinical characteristics and outcomes of the patients with SBP due to ESBL-EK are shown in Table 3 as a function of the effectiveness of the initial therapy. In the cases, the treatment failure rate and 30-day mortality rate of cases receiving effective initial therapy were significantly lower than those of patients receiving ineffective initial therapy (13% *vs*. 61%, *P *= .036).

**Table 3:** Characteristics and outcomes of patients with spontaneous bacterial peritonitis due to extended-spectrum β-lactamase-producing *Escherichia coli *and *Klebsiella *species (ESBL-EK), according to the effectiveness of the initial therapy

Characteristics	Initial therapy		*P*-value
		
	Effective (n = 8)	Ineffective (n = 18)	
Age (mean in year, SD)	62.1 ± 9.3	58.3 ± 10.4	0.363
Male (n, %)	4 (50%)	15 (83%)	0.149
*Escherichia coli *(n, %)	5 (63%)	12 (67%)	1.000
Child-Pugh score (mean, SD)	10.4 ± 1.6	11.1 ± 1.6	0.330
APACHE II score (mean, SD)	15.6 ± 3.8	15.6 ± 3.7	0.993

**Outcomes**			
Initial response^†^			
Complete response (n, %)	1 (13%)	1 (6%)	0.529
Treatment failure (n, %)	2 (25%)	13 (72%)	0.038
7-day mortality (n, %)	1 (13%)	8 (44%)	0.190
30-day mortality (n, %)	1 (13%)	11 (61%)	0.036

SD – standard deviation

APACHE II – Acute Physiologic and Chronic Health Evaluation II

^† ^Initial response was assessed 72 hours after the initiation of empirical treatment

### Risk factors for SBP due to ESBL-EK

The risk factors associated with SBP due to ESBL-EK are listed in Table 4. No patient among either the cases or controls had neutropenia, central venous catheter, immunosuppressive agents, polymicrobial infection, or invasive procedure within 72 hours. From a univariate analysis using conditional logistic regression, hospital stay (≥2 weeks) before onset of SBP, previous history of SBP and prior use of any antibiotics within 30 days were significant factors associated with infection by ESBL-EK.

**Table 4:** Risk factors for spontaneous bacterial peritonitis (SBP) due to extended-spectrum β-lactamase-producing *Escherichia coli *and *Klebsiella *species (ESBL-EK)

Risk factors	SBP due to		OR (95% CI)
		
	ESBL-EK (n = 26)	Non-ESBL-EK (n = 78)	
Hospital stay before onset of SBP	23.3 ± 24.8	1.6 ± 5.0	-
(mean in days, SD)			
≥ 2 weeks (n, %)	13 (50%)	3 (4%)	35.11 (4.57 to 269.72)
Presence of hepatocellular carcinoma (n, %)	9 (35%)	21 (27%)	1.46 (0.55 to 3.94)
Presentation with septic shock (n, %)	11 (42%)	22 (28%)	2.21 (0.78 to 6.31)
Presence of bacteremia (n, %)	5 (19%)	13 (17%)	1.16 (0.41 to 3.33)
ICU care (n, %)	5 (19%)	7 (9%)	2.53 (0.70 to 9.12)
Previous history of SBP (n, %)	19 (73%)	23 (30%)	12.91 (2.88 to 57.76)
Prior use of antibiotics within 30 days^† ^(n, %)	21 (81%)	13 (17%)	15.13 (4.44 to 51.52)

SD – standard deviation

OR – odds ratio

CI – confidence interval

^†^All the patients with a history of use of antibiotics had received 3^rd ^generation cephalosporins within 30 days before the onset of SBP.

## Discussion

In a previous study [16], we demonstrated that ESBL production adversely affected clinical outcomes in bacteremic SBP due to EK. However, because bacteremia is a more severe infection and can be an independent poor prognostic factor for SBP [17], we cannot extrapolate the results of the previous study to SBP without bacteremia. Therefore, we conducted the current study to evaluate the outcomes of SBP due to ESBL-EK based on isolation from ascites, compared with those of SBP due to non-ESBL-EK.

In patients with liver cirrhosis, Child-Pugh and APACHE II scores are known to be the most important factors influencing mortality due to SBP [1]. We tried to minimize the confounding effects of Child-Pugh and APACHE II scores by using matched controls. In addition we matched other potential factors influencing the outcome of SBP: age, gender, and species of infecting microorganism. Even after matching these five important confounders, we found that SBP due to ESBL-EK had poorer outcomes than SBP due to non-ESBL-EK.

Among the other factors influencing the outcomes of ESBL infection, the impact of a delay in effective initial treatment was controversial. Lautenbach *et al*. and our previous work showed that a delay in effective treatment for ESBL-producing organisms did not result in poorer clinical outcomes in patients with urinary tract and bloodstream infections generally originating from the pancreatobiliary tract [10,18]. In contrast, in patients with non-urinary infections, Hyle *et al*. demonstrated that inadequate initial therapy was an independent risk factor for mortality in ESBL-EK infections [19]. In the current study, there was no statistically significant effect of the five major confounders, and ineffective initial treatment was associated with higher initial treatment failure and 30-day mortality rate, despite the use of effective carbapenems once the reports of the susceptibility of the microorganisms had been received.

Therefore, it would seem reasonable to treat patients with SBP due to ESBL-EK with effective antibiotics straight away. However, SBP due to ESBL-EK accounted for only 7.5% (26/346) of the SBP due to EK, and the injudicious use of broad antimicrobial regimens is likely to result in further emergence of resistance. We suggest that one should use antibiotics active against ESBL-producing organisms in those selected patients who have a high risk of infection. The risk factors for the development of infection with ESBL-producing organisms that have been listed in previous studies are: length of hospital stay and ICU stay, presence of central venous catheter, prior administration of an antibiotic, and severity of illness etc [10,11,18,20,21]. In the current study, univariate analysis using conditional logistic regression showed that hospital stay (≥2 weeks) before onset of SBP, previous history of SBP and prior use of any antibiotics within 30 days were significantly associated factors for ESBL-EK infections. To promote the prudent use of antimicrobial agents, we need further efforts to identify the risk factors for ESBL infection, as well as to optimize initial therapy [22].

Our study has potential limitations. First, we did not evaluate the 'attributable' mortality of SBP; some deaths in our study may not have been related to the SBP. However, efforts to designate outcomes as 'attributable' to infection are often subjective and inconsistent [19,23]. We therefore employed unambiguous definitions, namely in-hospital mortality rates (7-day, 30-day) as the primary outcomes. Second, because the present study was retrospective and non-randomized, there was potential for confounding and bias due to unknown factors during the selection of patients and analysis of the results. However, all case and control patients were selected from the same microbiology laboratory in the same hospital, and they were matched according to the defined criteria. The potential for selection bias and confounding effects should therefore be small. Finally, the small sample size limited our capacity to identify risk factors for infection with ESBL-producing EK using multivariate analysis. Moreover the univariate analysis should be interpreted with caution because of possible confounding effect of unidentified risk factors.

## Conclusion

Our results indicated that SBP due to ESBL-EK had poorer outcomes than SBP due to non-ESBL-EK and 'ineffective initial therapy' may be a cause of the higher rates of treatment failure and mortality in SBP due to ESBL-EK. However, SBP due to ESBL-EK accounted for only 7.5% (26/346) of the SBP due to EK, and the imprudent use of broad spectrum antibiotics should be avoided. Therefore, studies aimed at identifying the risk factors for SBP caused by ESBL-producing organism are needed in order to optimize initial therapy for selected patients who have a high risk of infection.

## Competing interests

The authors declare that they have no competing interests.

## Authors' contributions

All authors conceived of the study. KHS and JHJ collected the data. KHS and NJK carried out data analysis and interpretation. WBP, SWP, HBK, MDO, HSL and KWC carried out data interpretation. KHS and NJK drafted the manuscript. All authors have read and approved the final manuscript.

## Pre-publication history

The pre-publication history for this paper can be accessed here:

http://www.biomedcentral.com/1471-2334/9/41/prepub

## References

[B1] Christou L Pappas G Falagas ME Bacterial infection-related morbidity and mortality in cirrhosis Am J Gastroenterol 2007 102 7 1510 1517 10.1111/j.1572-0241.2007.01286.x 17509025

[B2] Fernandez J Navasa M Gomez J Colmenero J Vila J Arroyo V Rodes J Bacterial infections in cirrhosis: epidemiological changes with invasive procedures and norfloxacin prophylaxis Hepatology 2002 35 1 140 148 10.1053/jhep.2002.30082 11786970

[B3] Rimola A Navasa M Arroyo V Experience with cefotaxime in the treatment of spontaneous bacterial peritonitis in cirrhosis Diagn Microbiol Infect Dis 1995 22 1–2 141 145 10.1016/0732-8893(95)00089-S 7587029

[B4] Franca A Giordano HM Seva-Pereira T Soares EC Five days of ceftriaxone to treat spontaneous bacterial peritonitis in cirrhotic patients J Gastroenterol 2002 37 2 119 122 10.1007/s005350200006 11871762

[B5] Hoefs JC Runyon BA Spontaneous bacterial peritonitis Dis Mon 1985 31 9 1 48 10.1016/0011-5029(85)90002-1 3899555

[B6] Felisart J Rimola A Arroyo V Perez-Ayuso RM Quintero E Gines P Rodes J Cefotaxime is more effective than is ampicillin-tobramycin in cirrhotics with severe infections Hepatology 1985 5 3 457 462 10.1002/hep.1840050319 3888810

[B7] Rimola A Salmeron JM Clemente G Rodrigo L Obrador A Miranda ML Guarner C Planas R Sola R Vargas V Two different dosages of cefotaxime in the treatment of spontaneous bacterial peritonitis in cirrhosis: results of a prospective, randomized, multicenter study Hepatology 1995 21 3 674 679 10.1002/hep.1840210312 7875666

[B8] Kim BN Woo JH Kim MN Ryu J Kim YS Clinical implications of extended-spectrum beta-lactamase-producing Klebsiella pneumoniae bacteraemia J Hosp Infect 2002 52 2 99 106 10.1053/jhin.2002.1288 12392901

[B9] Kim YK Pai H Lee HJ Park SE Choi EH Kim J Kim JH Kim EC Bloodstream infections by extended-spectrum beta-lactamase-producing Escherichia coli and Klebsiella pneumoniae in children: epidemiology and clinical outcome Antimicrob Agents Chemother 2002 46 5 1481 1491 11959586 10.1128/AAC.46.5.1481-1491.2002 PMC127143

[B10] Lautenbach E Patel JB Bilker WB Edelstein PH Fishman NO Extended-spectrum beta-lactamase-producing Escherichia coli and Klebsiella pneumoniae: risk factors for infection and impact of resistance on outcomes Clin Infect Dis 2001 32 8 1162 1171 10.1086/319757 11283805

[B11] Schiappa DA Hayden MK Matushek MG Hashemi FN Sullivan J Smith KY Miyashiro D Quinn JP Weinstein RA Trenholme GM Ceftazidime-resistant Klebsiella pneumoniae and Escherichia coli bloodstream infection: a case-control and molecular epidemiologic investigation J Infect Dis 1996 174 3 529 536 8769610 10.1093/infdis/174.3.529

[B12] Paterson DL Ko WC Von Gottberg A Mohapatra S Casellas JM Goossens H Mulazimoglu L Trenholme G Klugman KP Bonomo RA Antibiotic therapy for Klebsiella pneumoniae bacteremia: implications of production of extended-spectrum beta-lactamases Clin Infect Dis 2004 39 1 31 37 10.1086/420816 15206050

[B13] Infante-Rivard C Esnaola S Villeneuve JP Clinical and statistical validity of conventional prognostic factors in predicting short-term survival among cirrhotics Hepatology 1987 7 4 660 664 10.1002/hep.1840070408 3610046

[B14] Knaus WA Draper EA Wagner DP Zimmerman JE APACHE II: a severity of disease classification system Crit Care Med 1985 13 10 818 829 10.1097/00003246-198510000-00009 3928249

[B15] Rimola A Garcia-Tsao G Navasa M Piddock LJ Planas R Bernard B Inadomi JM Diagnosis, treatment and prophylaxis of spontaneous bacterial peritonitis: a consensus document. International Ascites Club J Hepatol 2000 32 1 142 153 10.1016/S0168-8278(00)80201-9 10673079

[B16] Kang CI Kim SH Park WB Lee KD Kim HB Oh MD Kim EC Lee HS Choe KW Clinical outcome of bacteremic spontaneous bacterial peritonitis due to extended-spectrum beta-lactamase-producing Escherichia coli and Klebsiella pneumoniae Korean J Intern Med 2004 19 3 160 164 15481607 10.3904/kjim.2004.19.3.160 PMC4531561

[B17] Cho JH Park KH Kim SH Bang JH Park WB Kim HB Kim NJ Oh MD Lee HS Choe KW Bacteremia is a prognostic factor for poor outcome in spontaneous bacterial peritonitis Scand J Infect Dis 2007 39 8 697 702 10.1080/00365540701299582 17654346

[B18] Kang CI Kim SH Kim DM Park WB Lee KD Kim HB Oh MD Kim EC Choe KW Risk factors for and clinical outcomes of bloodstream infections caused by extended-spectrum beta-lactamase-producing Klebsiella pneumoniae Infect Control Hosp Epidemiol 2004 25 10 860 867 10.1086/502310 15518030

[B19] Hyle EP Lipworth AD Zaoutis TE Nachamkin I Bilker WB Lautenbach E Impact of inadequate initial antimicrobial therapy on mortality in infections due to extended-spectrum beta-lactamase-producing enterobacteriaceae: variability by site of infection Arch Intern Med 2005 165 12 1375 1380 10.1001/archinte.165.12.1375 15983286

[B20] Paterson DL Ko WC Von Gottberg A Mohapatra S Casellas JM Goossens H Mulazimoglu L Trenholme G Klugman KP Bonomo RA International prospective study of Klebsiella pneumoniae bacteremia: implications of extended-spectrum beta-lactamase production in nosocomial Infections Ann Intern Med 2004 140 1 26 32 14706969 10.7326/0003-4819-140-1-200401060-00008

[B21] Jacoby GA Munoz-Price LS The new beta-lactamases N Engl J Med 2005 352 4 380 391 10.1056/NEJMra041359 15673804

[B22] Paterson DL Rice LB Empirical antibiotic choice for the seriously ill patient: are minimization of selection of resistant organisms and maximization of individual outcome mutually exclusive? Clin Infect Dis 2003 36 8 1006 1012 10.1086/374243 12684913

[B23] Cosgrove SE Carmeli Y The impact of antimicrobial resistance on health and economic outcomes Clin Infect Dis 2003 36 11 1433 1437 10.1086/375081 12766839

